# Digital Twin–Assisted Risk Disclosure in Adults Undergoing Elective Bronchoscopy: Multicenter Randomized Controlled Trial

**DOI:** 10.2196/81382

**Published:** 2026-10-06

**Authors:** Mingming Deng, Weidong Xu, Fei Tang, Hong Chen, Zhen Yang, Feng Wang, Nan Zhang, Haihong Wu, Jia Li, Ziwen Zheng, Sinan Wu, Gang Hou

**Affiliations:** 1 National Center for Respiratory Medicine, State Key Laboratory of Respiratory Health and Multimorbidity, National Clinical Research Center for Respiratory Diseases, Institute of Respiratory Medicine, Chinese Academy of Medical Sciences, Department of Pulmonary and Critical Care Medicine, Center of Respiratory Medicine, China-Japan Friendship Hospital, 2 Yinghuayuan East Street, Chaoyang District, Beijing, 100029, China, 86 10 84205729; 2 China-Japan Friendship Hospital (Institute of Clinical Medical Sciences), Chinese Academy of Medical Sciences & Peking Union Medical College, Beijing, China; 3 Department of Interventional Pulmonology and Endoscopic Diagnosis and Treatment Center, Anhui Chest Hospital, Hefei, China; 4 Department of Pulmonary and Critical Care Medicine, Second Affiliated Hospital of Harbin Medical University, Harbin, China; 5 Department of Respiratory and Critical Medicine, First Medical Center, Chinese PLA General Hospital, Beijing, China; 6 Department of Respiratory and Critical Care Medicine, Beijing Institute of Respiratory Medicine, Beijing Chao-Yang Hospital, Capital Medical University, Beijing, China; 7 Department of Pulmonary and Critical Care Medicine, Emergency General Hospital, Beijing, China; 8 Department of Pulmonary and Critical Care Medicine, Hainan Affiliated Hospital of Hainan Medical University, Hainan General Hospital, Hainan, China; 9 National Center for Respiratory Medicine, State Key Laboratory of Respiratory Health and Multimorbidity, National Clinical Research Center for Respiratory Diseases, Institute of Respiratory Medicine, Chinese Academy of Medical Sciences, Department of Clinical Research and Data Management, Center of Respiratory Medicine, China-Japan Friendship Hospital, Beijing, China

**Keywords:** anxiety, bronchoscopy, digital-twin, simulator, informed consent

## Abstract

**Background:**

Risk disclosure before bronchoscopy should provide sufficient information for informed consent, but detailed text-based risk disclosure may increase procedural anxiety. Patient-specific visualization with a digital twin–based bronchoscopy simulator may help patients understand bronchoscopy and its risks in a more individualized manner.

**Objective:**

This study evaluated whether digital twin–assisted risk disclosure reduces prebronchoscopy anxiety and improves postbronchoscopy satisfaction compared with conventional risk disclosure in adults scheduled for elective bronchoscopy.

**Methods:**

We conducted a multicenter, parallel-group randomized controlled trial. Adults aged 18 years or older scheduled for elective bronchoscopy under local anesthesia were included. Participants were randomized to either a digital-twin informed-consent group, which received standard written information plus a physician-led oral explanation supported by a patient-specific simulator visualization, or a conventional informed-consent group, which received the same written information plus a standard physician-led oral explanation without simulator visualization. Owing to the nature of the intervention, participants and physicians were not blinded. The primary outcome was the change in self-reported anxiety after risk disclosure, measured using the visual analog scale (VAS) and the modified Amsterdam Preoperative Anxiety and Information Scale (APAIS). Linear mixed models with a group-by-time interaction were used for the main analysis. The secondary outcome was postbronchoscopy satisfaction.

**Results:**

Of 150 patients assessed for eligibility, 122 were randomized and analyzed, with 61 participants in each group. Compared with conventional risk disclosure, digital twin–assisted disclosure produced greater reductions in anxiety on the VAS (group by time β=−15.89, SE 3.08, 95% CI −21.99 to −9.78; *P*<.001) and APAIS total anxiety score (β=−6.77, SE 0.98, 95% CI −8.71 to −4.83; *P*<.001). Similar effects were observed for APAIS procedure-related anxiety (β=−4.25, 95% CI −5.47 to −3.02; *P*<.001) and APAIS outcome-related anxiety (β=−2.52, 95% CI −3.46 to −1.59; *P*<.001). Clinically meaningful improvement occurred more often in the digital-twin group for VAS (30/61, 49.2% vs 4/61, 6.6%) and APAIS (33/61, 54.1% vs 6/61, 9.8%; both *P*<.001). Satisfaction was higher in the digital-twin group (mean 16.89, SD 2.08 vs mean 14.38, SD 1.89; *P*<.001). All participants completed bronchoscopy without complications or adverse conditions.

**Conclusions:**

Patient-specific digital twin–visualization during physician-led risk disclosure reduced short-term self-reported anxiety and modestly improved satisfaction. The innovation lies in using each patient’s computed tomography–derived airway and lesion anatomy during consent rather than standardized text, audiovisual content, or graphic narratives evaluated previously. This multicenter trial extends digital-twin technology from bronchoscopy training to individualized risk communication. In clinical practice, the approach could supplement physician-led consent in units with computed tomography and simulator infrastructure; however, time-matched studies should establish objective benefits, workflow burden, cost-effectiveness, accessibility, and applicability to highly anxious or resource-limited populations before wider adoption.

## Introduction

### Background and Rationale

Informed consent before bronchoscopy is both an ethical requirement and a clinically consequential communication process. Valid consent requires that patients understand the indication, expected course, material benefits and harms, and reasonable alternatives, while retaining an opportunity to ask questions and decide voluntarily [1]. This is particularly challenging in elective bronchoscopy because many patients remain conscious or lightly sedated and must anticipate unfamiliar airway sensations, cough, breathlessness, bleeding, procedural complications, and uncertainty about diagnostic findings. Anxiety and discomfort are prominent features of the bronchoscopy experience and are associated with lower satisfaction and reluctance to undergo the procedure again [2]. Clinicians must, therefore, disclose material risks adequately without allowing an undifferentiated list of complications to amplify fear.

Conventional consent usually combines a written form with face-to-face explanation. Written materials standardize content and provide a durable record, whereas oral discussion permits questions and adaptation to individual concerns. However, dense terminology, numerical risk information, and variable health literacy can impair comprehension, while oral delivery may vary among clinicians and be constrained by time. A recent systematic review found that adding more or more specific risk information could improve knowledge but often increased anxiety, whereas lower readability and verbal risk presentation sometimes improved patient-centered outcomes [3]. Another review found that interactive digital interventions and verbal test-and-feedback or teach-back approaches were more consistently associated with comprehension gains than static written or audiovisual materials, although intervention heterogeneity and risk of bias were substantial [4]. Visual risk communication may assist some patients, but its effectiveness depends on framing, numeracy, and graph literacy [5].

Multimedia and electronic consent can integrate text, narration, animation, and user-controlled review, thereby improving consistency and allowing patients to revisit information. In a systematic review of 40 surgical randomized controlled trials (RCTs), digital consent improved early comprehension in 21 of 30 studies, but most trials found no difference in anxiety or satisfaction, and feasibility was infrequently evaluated [1]. A separate review of electronic consent, conducted largely in research-enrollment settings, found improved or noninferior comprehension, acceptability, and usability, but sometimes longer completion times and limited evidence regarding workload or participant retention [6]. In endoscopy, a colonoscopy RCT similarly showed improved knowledge retention and satisfaction with a supplemental consent video, without establishing an anxiolytic effect [7]. Digital delivery should therefore not be equated with anxiety reduction; content, timing, interactivity, accessibility, and clinician involvement remain important.

Randomized evidence from bronchoscopy and endoscopy illustrates this inconsistency. Detailed written risk disclosure before bronchoscopy increased periprocedural anxiety [8], whereas a multimedia supplement reduced anxiety and improved satisfaction in another trial [9]. A procedure-specific graphic narrative improved satisfaction with bronchoscopy consent but did not change anxiety [10]. During bronchoscopy, virtual-reality distraction using nature scenes and music reduced postprocedural anxiety in 1 trial [11]. By contrast, virtual-reality hypnosis produced no overall improvement in anxiety or tolerance [12], and a 2025 music trial improved comfort but not anxiety [13]. A later RCT found that virtual reality–guided relaxation before and during bronchoscopy reduced anxiety and physiological arousal [14]. In upper gastrointestinal endoscopy, preprocedural virtual-reality education improved information recall but not anxiety [15], while a 2026 trial of virtual-reality distraction during unsedated gastroscopy found no reduction in anxiety, pain, or discomfort [16]. A systematic review of awake invasive procedures also found generally favorable effects of virtual reality on subjective anxiety and pain, but effects on physiological indicators and additional sedation or analgesia remained unclear [17]. Importantly, distraction and relaxation interventions act through mechanisms different from risk communication and should not be treated as equivalent consent strategies.

A further distinction is whether visual information is standardized or patient specific. Videos, illustrations, and generic 3D scenes present essentially the same anatomy and procedural sequence to every patient. A model reconstructed from the patient’s own computed tomography (CT) images can instead anchor the discussion to that individual’s bronchial tree, lesion location, and planned bronchoscopic route. This may reduce the mental effort required to translate abstract risk descriptions into personally relevant anatomy and may enable more focused questions. Evidence from other specialties supports the informational value of personalization but does not demonstrate an automatic anxiety benefit. In randomized neurosurgical studies, patient-specific immersive 3D or augmented-reality visualization improved objective understanding and/or satisfaction, whereas between-group differences in anxiety change were not significant [18,19]. Anatomical personalization may therefore improve how information is represented and discussed, but its effect on anxiety remains uncertain.

Digital-twin terminology remains heterogeneous. Recent reviews broadly describe a patient digital twin as a viewable, patient-specific digital replica that can inform decisions and distinguish one-time simulation twins from continuously updated monitoring twins [20,21]. Within this framework, the present platform is best characterized as a CT-derived simulation patient digital twin rather than a continuously updating predictive twin. Training with varied CT-derived bronchial models on this simulator platform has improved novices’ transfer to unfamiliar anatomy and skill retention [22]. However, evidence of educational effectiveness for clinicians does not establish a benefit when the platform is repurposed for patient-facing risk disclosure.

### Objectives

We reasoned that physician-guided visualization of a patient’s own airway, lesion, and intended bronchoscopic route could convert generic procedural information into a more concrete mental model, facilitate focused questions, and reduce uncertainty about what would occur during bronchoscopy. This mechanism remained hypothetical; vivid visualization could also increase distress, and additional clinician attention or exposure time could influence outcomes independently of anatomical personalization. Existing bronchoscopy RCTs had not evaluated patient-specific CT-derived simulation as part of risk disclosure. We therefore conducted a multicenter RCT comparing conventional risk disclosure with digital twin–assisted, physician-led disclosure in adults undergoing elective bronchoscopy. We hypothesized that the patient-specific approach would produce a greater immediate reduction in self-reported anxiety, assessed using the visual analog scale (VAS) and the modified Amsterdam Preoperative Anxiety and Information Scale (APAIS), and would improve postprocedural satisfaction. The trial evaluated the clinical effect of the communication strategy; it did not directly measure comprehension or perceived uncertainty.

## Methods

### Trial Design

This was a multicenter, parallel-group RCT with individual allocation in a 1:1 ratio. Participants were assigned to either digital twin–assisted disclosure (written risk information plus patient-specific simulator visualization) or conventional disclosure (the same written information plus physician-led oral explanation without simulator visualization).

### Changes to the Trial Protocol

No changes were made to the eligibility criteria, interventions, or outcome definitions after trial commencement. The original protocol proposed analysis of covariance (ANCOVA) or change-score comparisons; in response to peer review, the repeated anxiety outcomes were reanalyzed using linear mixed-effects models to account for within-participant correlation. This analytic refinement was post hoc. This trial is reported in accordance with the CONSORT (Consolidated Standards of Reporting Trials) 2025 statement [23]. Because the intervention incorporated an interactive digital health technology, applicable items from the CONSORT-EHEALTH (Consolidated Standards of Reporting Trials of Electronic and Mobile Health Applications and Online Telehealth) extension were also followed [24]. The completed CONSORT 2025 (Checklist 1) and CONSORT-EHEALTH (Checklist 2) checklists are provided as supplementary files.

### Trial Setting

The trial was conducted at 3 hospitals in China from June to July 2024.

### Trial Registration

The trial was prospectively registered at ClinicalTrials.gov (NCT06441149) on May 29, 2024, before recruitment began in June 2024.

### Protocol and Statistical Analysis Plan

The trial protocol and statistical analysis plan may be obtained from the corresponding author upon reasonable request.

### Eligibility Criteria

We included patients aged 18 years or older who required elective bronchoscopy under local anesthesia for pulmonary disease. We excluded individuals with substantial communication impairment due to dementia, impaired verbal communication, hearing dysfunction, or illiteracy. All patients admitted to the clinics were screened for eligibility and recruited consecutively from June to July 2024.

### Sequence Generation

Simple randomization was used with a 1:1 allocation ratio and no blocking, stratification, or other restriction. An independent statistician generated the allocation sequence, and a second statistician uploaded it to the research data-collection system.

### Allocation Concealment Mechanism

Group assignment was released only after enrollment, thereby concealing the allocation sequence until the intervention assignment.

### Implementation

Personnel enrolling participants and physicians delivering the disclosure interventions did not have access to the allocation list before assignment.

### Blinding

Participants and physicians delivering the intervention could not be blinded because the simulator visualization was evident. Administrative study staff who distributed and collected baseline and follow-up questionnaires were blinded to group assignment and were not involved in consent delivery, bronchoscopy, or statistical analysis.

### Intervention and Comparator

#### Intervention Delivery and Fidelity

Intervention reporting was structured according to the TIDieR (Template for Intervention Description and Replication; Checklist 3) [25]. The core components shared by both groups were standardized written risk information, one face-to-face prebronchoscopy discussion, and the opportunity to ask questions; the digital-twin group additionally received patient-specific CT-based visualization. At each participating bronchoscopy unit, 1 bronchoscopy physician trained in a uniform disclosure protocol delivered the assigned format. The combined preprocedural information process, including review of written materials, typically occupied approximately 30 to 40 minutes in routine workflow; exact group-specific physician-interaction and simulator-viewing times were not prospectively timed. All participants received their assigned single session. Delivery was not formally audited using recordings, independent ratings, or prospectively completed adherence checklists, and no protocol modifications were made during the trial.

#### Conventional Risk Disclosure

The physician reviewed the written information, explained the planned bronchoscopy and associated risks face-to-face, and answered questions. No simulator, app, or patient-specific visualization was used. Anxiety was reassessed immediately after the session.

#### Digital Twin–Assisted Risk Disclosure

The intervention used the previously described [22] digital-twin bronchoscopy simulator (Simulation AI Plus, Zhejiang UE Medical Corp). Before the face-to-face session, the participant’s chest CT data were preloaded and processed using CT preprocessing, airway segmentation, 3D mesh reconstruction, centerline extraction, and virtual bronchoscope navigation to generate a patient-specific bronchial tree, airway-lesion relationship, and simulated bronchoscopic route. The physician controlled the patient-facing display, oriented the participant to the CT-derived anatomy and planned route, explained expected procedural steps and associated risks in that individualized anatomical context, and answered questions. Participants viewed but did not independently operate the simulator. Anxiety was reassessed immediately after the session (Figure 1).

The upper section of the flowchart illustrates the randomization of patients who met the inclusion criteria into 2 groups: 1 group received informed consent for bronchoscopy using the conventional method, whereas the other group received additional visualization via a bronchoscopy simulator. The lower section shows the preimported CT data of the patient reconstructed into the tracheal tree, in which a trained bronchoscopy physician performed a bronchoscopy simulation using the digital twin–based bronchoscopy simulator.

During the bronchoscopy, approximately 5 milliliters of 2% lidocaine gel was applied to the patient’s nasal cavity and introduced into the bronchoscope. Subsequently, four 2-milliliter aliquots of 1% lidocaine solution were administered through the bronchoscope onto the vocal cords, the rongeur, and the 2 main bronchi. Additional lidocaine was administered as needed, based on the patient’s response.

After bronchoscopy, the clerical staff in the bronchoscopy unit distributed a postoperative-satisfaction questionnaire to all subjects. It comprised 4 questions answered using a 5-point Likert scale ranging from “strongly disagree” to “strongly agree.” Responses were recoded during the statistical analysis to ensure that higher scores indicated greater satisfaction.

**Figure 1.**
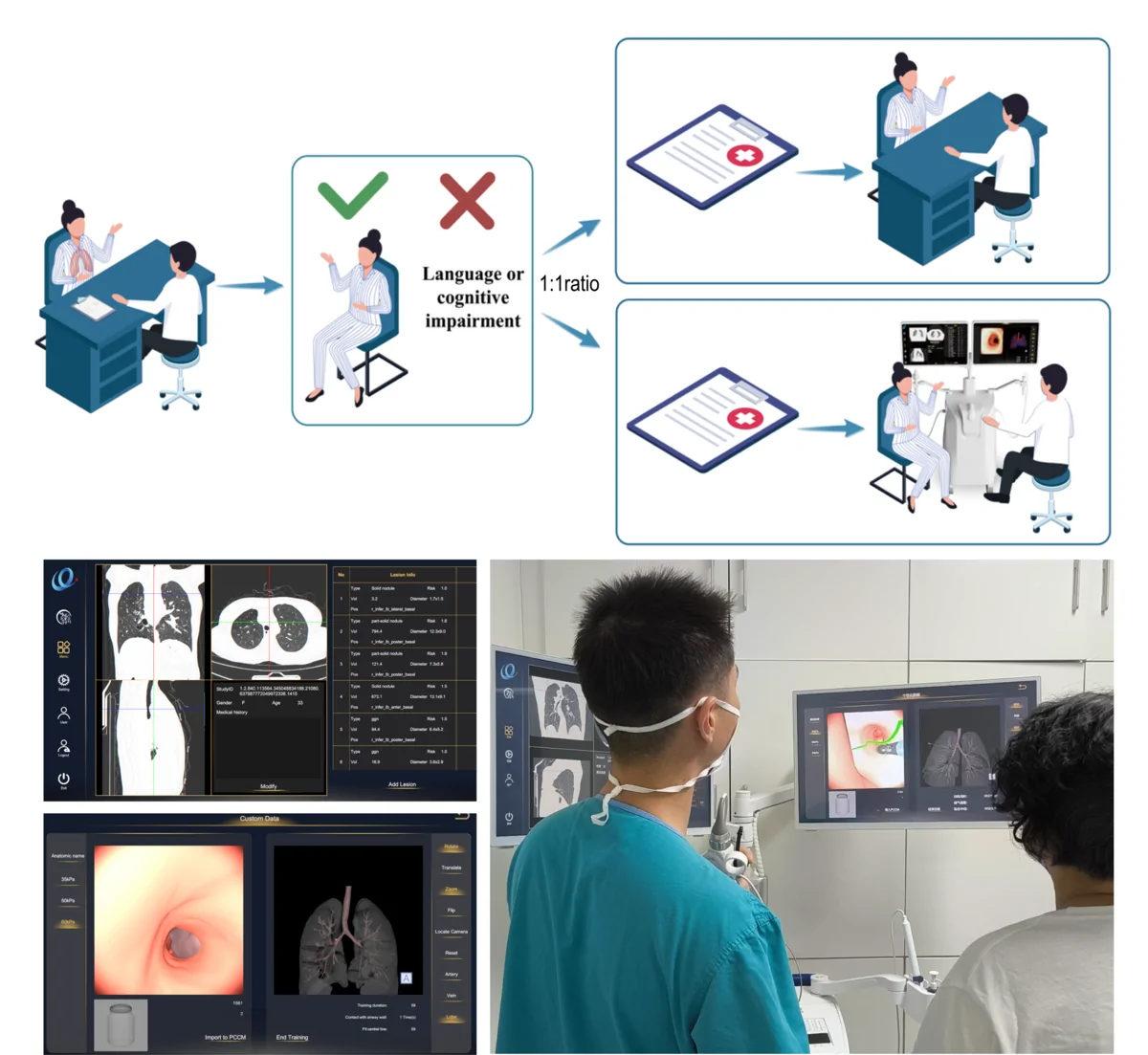
Study design flowchart.

### Outcomes

Baseline data included demographics, indications for bronchoscopy, a history of bronchoscopy, and whether the patient had previously received a preoperative explanation toward the provision of informed consent.

The main outcomes were anxiety levels (APAIS [26] and VAS [27] scores) at baseline and follow-up. The modified APAIS used six 5-point Likert items. The first three items assessed concerns and information needs regarding the results of the bronchoscopy examination: (1) “I am very worried about the results of this examination,” (2) “The results of this examination are constantly on my mind,” and (3) “I would like to know as much as possible about the results of this examination.” The last three items assessed concerns and information needs regarding the procedure: (4) “I am very worried about the procedure,” (5) “The procedure is constantly on my mind,” and (6) “I would like to know as much as possible about the procedure.”

The primary outcome was the change in anxiety levels after risk disclosure. The prespecified secondary outcome was satisfaction after bronchoscopy. According to a previous study, a difference of 8% of the theoretical range of a health-related quality of life tool is considered a minimal clinically important difference (MCID) [8]. Thus, for our primary outcome measures, a change of 8 mm in the VAS or a change of 2.4 points in the APAIS score was defined as clinically significant.

The VAS score was represented by a 100-mm line accompanied by vivid images for annotation. A line of 0 mm indicates no anxiety, whereas a line of 100 mm indicates severe anxiety. Every 20 mm was supplemented with an image representing the anxiety level, allowing patients to rate their anxiety levels more accurately.

The APAIS score consists of 6 questions, each rated on a scale of 1 to 5, with higher scores indicating greater interest or concern regarding the respective question. Considering the diverse spectrum of patients’ diseases, our questionnaire design incorporated 3 questions about the results of the intervention and 3 questions about the surgical process.

### Harms

Adverse events were defined as any unfavorable medical event occurring after the study intervention, irrespective of attribution. Events were graded as mild, moderate, or severe and recorded in the case-report form; serious adverse events were to be reported promptly to the sponsor and the ethics committee. Bronchoscopy-related complications and unintended effects were summarized by group.

### Sample Size

The sample size was calculated using PASS 2021 (NCSS, LLC) with a 2-tailed sample *t* test assuming equal variances. Based on prior bronchoscopy risk-disclosure literature [8], the assumed SD of the VAS anxiety score was approximately 15 points, and the clinically meaningful between-group difference was set at 8 points. This corresponds to a standardized effect size of approximately 0.53 (8/15), which represents a moderate target effect for planning purposes. With a 2-sided α of 0.05, 80% power, and 1:1 allocation, the required total sample size was 114 participants, corresponding to 57 per group. The original calculation did not include an attrition adjustment. The final randomized sample of 122 exceeded the calculated requirement of 114 by 8 (7.0%) participants.

### Statistical Methods

The repeated VAS and modified APAIS outcomes were reanalyzed using linear mixed-effects models fitted by restricted maximum likelihood. Each model included group, time, and the group-by-time interaction as fixed effects and a participant-level random intercept; denominator df were estimated using the Satterthwaite method. The interaction coefficient estimated the between-group difference in change from baseline. This post hoc reanalysis was conducted in response to peer review and superseded the protocol’s ANCOVA or change-score approach for the revised report. The primary analysis followed the intention-to-treat principle and included all 122 randomized participants in their assigned groups. Because every randomized participant received the allocated intervention, completed both assessments, and was analyzed, the per-protocol population was identical to the intention-to-treat population (n=61 per group); no separate per-protocol estimates or missing-data imputation were required. No formal multiplicity adjustment was applied to secondary or subscale analyses, which were interpreted as supportive and exploratory. No interim analyses or trial-level stopping guidelines were prespecified or performed.

Statistical analysis was conducted using SPSS Statistics software (version 24.0; IBM Corp). The Pearson correlation coefficient was used to examine the association between continuous variables. Chi-square tests were used to analyze relationships between categorical variables. For continuous variables, one-way ANOVA and post hoc Tukey multiple-comparison tests (for normally distributed data) or Kruskal-Wallis tests (for nonnormally distributed data) were used to assess differences among 3 or more groups. The 2-tailed *t* test (for normally distributed data) or Mann-Whitney *U* test (for nonnormally distributed data) was used to evaluate differences between 2 groups. A *P* value <.05 was considered statistically significant.

### Ethical Considerations

This study was conducted from June to July 2024 and was approved by the ethics committees at the China-Japan Friendship Hospital (2024-KY-095), the Second Affiliated Hospital of Harbin Medical University (KY2024-122), and the Anhui Chest Hospital (KJ2024-032). The study was registered at ClinicalTrials.gov (NCT06441149). All participants provided written informed consent before enrollment. No identifiable patient images were included; therefore, separate consent for publication was not required. Study data were deidentified and stored under restricted-access procedures. Participants received no compensation for participation. The data dictionary and statistical code have not been deposited in a public repository; requests for these materials can be directed to the corresponding author. Patients and members of the public were not involved in the design, conduct, reporting, or dissemination planning of this trial.

## Results

### Participant Flow, Including a Flow Diagram

Figure 2 illustrates participant flow. Of 150 patients assessed for eligibility, 28 were excluded: 6 because of hearing dysfunction, 6 because of illiteracy, and 16 because the anesthesia method changed before bronchoscopy. The remaining 122 participants were randomly assigned to conventional disclosure (n=61) or digital twin–assisted disclosure (n=61).

**Figure 2.**
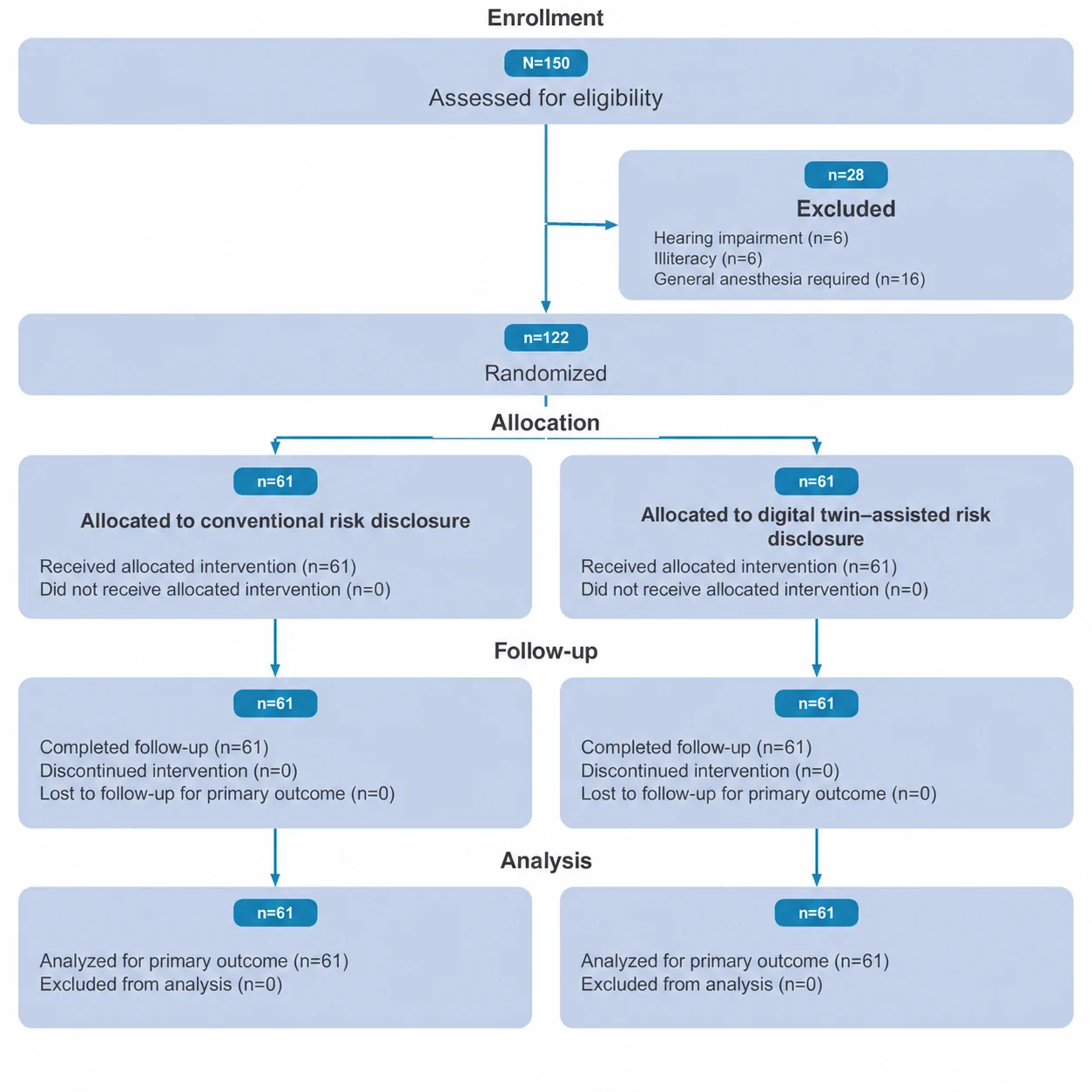
CONSORT (Consolidated Standards of Reporting Trials) 2025 (Checklist 1) participant flow diagram for a multicenter randomized controlled trial conducted in 3 Chinese hospitals from June to July 2024, comparing conventional risk disclosure with digital twin–assisted risk disclosure before elective bronchoscopy under local anesthesia.

### Recruitment

Recruitment occurred from June 14 to July 30, 2024, and the final follow-up was completed on August 15, 2024. The trial ended after completion of the planned follow-up and was not stopped early.

### Intervention and Comparator Delivery

All 122 randomized participants received their allocated disclosure intervention and completed follow-up; intervention delivery was not formally audited.

### Baseline Data

Baseline demographic and clinical characteristics were comparable between groups. Suspected pulmonary malignancy was the bronchoscopy indication in 32 of 61 (52.5%) participants in the conventional group and 28 of 61 (45.9%) participants in the digital-twin group. Mean baseline VAS scores were 23.11 (SD 22.92) and 23.54 (SD 21.42), respectively; corresponding modified APAIS total scores were 13.31 (SD 6.98) and 12.59 (SD 5.88; Table 1).

Baseline VAS scores were strongly correlated with baseline modified APAIS scores (Spearman ρ=0.648, *P*<.001). Multinomial logistic regression assessment showed that neither patient age nor a history of bronchoscopy correlated with baseline anxiety levels. However, baseline VAS scores were significantly higher in women than in men (mean 33.30, SD 24.28 vs mean 17.71, SD 18.67; *P*<.001).

**Table 1.** Baseline characteristics of patients.

Variables	Total (N=122)	Conventional informed-consent group (n=61)	Digital-twin informed-consent group (n=61)	Statistic	*P* value
Age (y), median (IQR)	59 (52-67)	61 (53-70)	57 (51-64)	–1.40^a^	.16
Sex (male), n (%)	78 (63.93)	41 (67.21)	37 (60.66)	0.57 (1)^b^	.45
Suspected pulmonary malignancy, n (%)	60 (49.18)	32 (52.46)	28 (45.90)	0.52 (1)^b^	.47
Prior discussion, n (%)	60 (49.18)	32 (52.46)	28 (45.90)	0.52 (1)^b^	.47
Prior bronchoscopy, n (%)	50 (40.98)	28 (45.90)	22 (36.07)	1.22 (1)^b^	.23
Anxiety VAS^c^ score, mean (SD)	23.33 (22.09)	23.11 (22.92)	23.54 (21.42)	–0.11 (120)^d^	.92
APAIS^e^ outcome score^f^, mean (SD)	6.70 (3.44)	6.66 (3.64)	6.74 (3.25)	–0.13 (120)^d^	.90
APAIS procedure score^g^, mean (SD)	6.25 (3.60)	6.66 (3.88)	5.85 (3.28)	1.24 (120)^d^	.23
APAIS anxiety total score^h^, mean (SD)	12.95 (6.43)	13.31 (6.98)	12.59 (5.88)	0.62 (120)^d^	.54

a Mann-Whitney *U* test.

b Chi-square test (*df*).

c VAS: visual analog scale.

d 2-tailed *t* test (*df*).

e APAIS: Amsterdam Preoperative Anxiety and Information Scale.

f APAIS outcome score=sum of 3 items related to anxiety about expected examination results.

g APAIS procedure score=sum of 3 items related to anxiety about the bronchoscopy process.

h APAIS total anxiety score=sum of all 6 modified APAIS items.

### Numbers Analyzed, Outcomes, and Estimation

Linear mixed-model analyses showed significant group-by-time interactions favoring the digital-twin group for VAS anxiety (β=−15.89, SE 3.08, 95% CI −21.99 to −9.78; *P*<.001), modified APAIS total score (β=−6.77, SE 0.98, 95% CI -8.71 to −4.83; *P*<.001), procedure subscore (β=−4.25, SE 0.62, 95% CI −5.47 to −3.02; *P*<.001), and outcome subscore (β=−2.52, SE 0.47, 95% CI −3.46 to −1.59; *P*<.001). The digital-twin informed-consent group exhibited a decrease in anxiety levels after the discussion, with changes in the VAS (mean −9.02, SD 14.23, 95% CI −12.66 to −5.37), APAIS total (mean −5.03, SD 5.90, 95% CI −6.54 to −3.52), APAIS procedure (median −2, IQR −5.5 to 0; range −11 to 3), and APAIS outcome (median 0, IQR −4 to 0; range −12 to 3) scores. Significantly larger decreases were observed in all 4 of these scores in the digital-twin informed-consent group compared with the scores in the conventional informed-consent group.

The digital-twin informed-consent group exhibited a decrease in anxiety levels after the discussion, with 49.2% (30/61) of patients reaching the MCID on the VAS, compared to 6.6% (4/61) in the conventional informed-consent group (*P*<.001; Table 2). Similarly, 54.1% (33/61) of patients in the digital-twin informed-consent group reached the MCID on the APAIS, compared to 9.8% (6/61) in the conventional group (*P*<.001).Relative risks for achieving the MCID (digital-twin vs conventional) were 7.50 (95% CI 2.81‐20.00) for VAS, 5.50 (95% CI 2.49‐12.17) for the modified APAIS total score, 3.20 (95% CI 1.73‐5.92) for the procedure subscore, and 3.38 (95% CI 1.67‐6.83) for the outcome subscore.

Although both groups reported high satisfaction, the digital-twin group had a modestly higher satisfaction score than the conventional group (mean 16.89, SD 2.08 vs mean 14.38, SD 1.89; *P*<.001). Participants receiving digital twin–assisted disclosure were more likely to judge the risk information as appropriate and were less likely to report that the information was worrying (Table 3).

**Table 2.** Changes in outcome measures at follow-up assessment.

Variables	Total (N=122)	Conventional informed-consent group (n=61)	Digital-twin informed-consent group (n=61)	Statistic	*P* value
Changes in outcome measures at follow-up assessment					<.001
Anxiety VAS^a^ score, mean (95% CI)	122	7.52 (3.17 to 11.88)	–9.02 (–12.66 to –5.37)	5.827 (120)^b^	
APAIS^c^ procedure score^d^ (median, IQR, range)	122	0 (0 to 3; −4 to 12)	−2 (−5.5 to 0; −11 to 3)	–4.881^e^	
APAIS outcome score^f^, mean (95% CI)	122	0 (−4 to 6)	0 (−12 to 3)	–5.942^e^	
APAIS total anxiety score^g^, mean (95% CI)	122	1.93 (0.68 to 3.19)	–5.03 (–6.54 to –3.52)	7.095 (120)^b^	
The proportion of each group achieving the mean change in MCID^h^ scores					<.001
APAIS total response rate, n (%)	39 (31.9)	6 (9.8)	33 (54.1)	27.48 (1)^i^	
APAIS outcome response rate, n (%)	35 (28.6)	8 (13.1)	27 (44.3)	14.46 (1)^i^	
APAIS procedure response rate, n (%)	42 (34.4)	10 (16.4)	32 (52.5)	17.57 (1)^i^	
VAS response rate, n (%)	34 (27.8)	4 (6.6)	30 (49.2)	27.56 (1)^i^	

a VAS: visual analog scale.

b 2-tailed *t* test (*df*).

c APAIS: Amsterdam Preoperative Anxiety and Information Scale.

d APAIS procedure score=sum of 3 items related to anxiety about the bronchoscopy process.

e Mann-Whitney *U* test.

f APAIS outcome score=sum of 3 items related to anxiety about expected examination results.

g APAIS total anxiety score=sum of all 6 modified APAIS items.

h MCID: minimal clinically important difference.

i Chi-square test (*df*).

**Table 3.** Responses to postprocedure satisfaction questionnaire.

Questionnaire statement	Number of percentage reporting moderate or strong agreement		*P* value^a^
	Conventional informed-consent group (n=61), n (%)	Digital-twin informed-consent group (n=61), n (%)	
I received as much information as I needed to make a decision regarding bronchoscopy	61 (100)	61 (100)	>.99
I received too much information regarding complications of bronchoscopy	45 (73.8)	19 (31.1)	.008
The information I received about bronchoscopy was helpful	60 (98.4)	60 (98.4)	>.99
The information I received about bronchoscopy worried me	39 (63.9)	19 (31.1)	.03

a *P* values computed using *χ*2 test.

### Ancillary Analyses

Significant differences in the regression slopes for the baseline and follow-up VAS and APAIS total scores were observed between the 2 groups (Figure 3). Notably, patients with higher anxiety levels at enrollment exhibited greater differences. These differences were statistically significant (*P*<.001), which violated the assumption of homogeneity of regression slopes required for the ANCOVA.

The regression slopes for baseline and follow-up VAS scores significantly different between the 2 groups. These differences were more pronounced among individuals with higher anxiety levels at enrollment.

**Figure 3.**
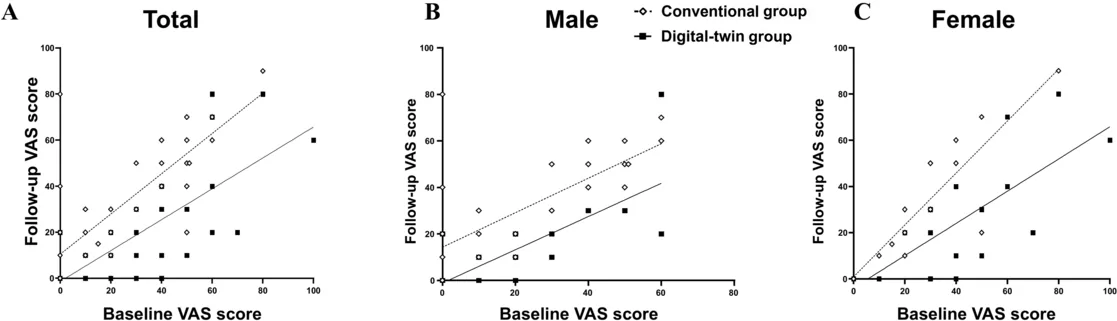
Scatter plot of baseline and follow-up anxiety visual analog scale (VAS) scores.

## Discussion

### Principal Findings

In this multicenter RCT, adding patient-specific CT-derived bronchoscopy visualization to physician-led risk disclosure produced larger immediate reductions in self-reported anxiety than conventional disclosure. The estimated between-group difference in change was 15.89 points for the VAS and 6.77 points for the modified APAIS total score, with concordant effects on procedure-related and outcome-related subscores. Clinically meaningful improvement, as operationally defined in this study, was also more frequent in the digital-twin group for the VAS (30/61, 49.2% vs 4/61, 6.6%) and modified APAIS total score (33/61, 54.1% vs 6/61, 9.8%). Postbronchoscopy satisfaction was modestly higher, and fewer participants described the information as excessive or worrying. These findings support an effect on the immediate emotional experience of risk disclosure, but they do not establish improved comprehension or downstream clinical benefit.

Clinical significance should be interpreted in context. Mean baseline VAS scores were approximately 23 on a 100-point scale, creating a possible floor effect. Nevertheless, the 15.89-point between-group difference exceeded the 8-point difference used for sample-size planning, and responder analyses were consistent across both instruments. However, the responder thresholds were derived from 8% of each instrument’s theoretical range rather than a bronchoscopy-specific validation study. They therefore provide supportive, not definitive, evidence of clinical importance. Satisfaction was already high in both groups, so its modest increase may also reflect a ceiling effect.

### Comparison With Previous Evidence

The findings are consistent with evidence that the way risks are communicated can alter patients’ emotional responses. In the conventional group, mean VAS anxiety increased by 7.52 (95% CI 3.17-11.88) points after disclosure, whereas it decreased by 9.02 (95% CI −12.66 to −5.37) points in the digital-twin group. Uzbeck et al [8] found that detailed written disclosure before bronchoscopy increased anxiety, and a recent systematic review similarly concluded that more or more specific risk information may improve knowledge while increasing anxiety [3]. Both groups in our trial received the same standardized written information. The contrasting responses, therefore, suggest that the context in which risks are explained matters, although the design cannot separate anatomical personalization from visual presentation, clinician attention, or exposure time.

The results extend the broader digital-consent literature. Systematic reviews indicate that multimedia and electronic consent can improve early comprehension or usability, but most trials have not demonstrated consistent anxiety reductions [1,4,6]. In endoscopy, supplemental video consent improved knowledge retention and satisfaction [7], while a bronchoscopy graphic narrative improved satisfaction without reducing anxiety [10]. Trials of virtual-reality distraction or relaxation around bronchoscopy and upper gastrointestinal endoscopy have also produced positive and null anxiety findings [11-17]. These interventions mainly used standardized content or distraction. The present intervention instead linked risk discussion to each participant’s reconstructed airway, lesion, and planned route. This distinction may be important, but patient-specific communication, generic visualization, and immersive distraction should not be treated as equivalent mechanisms.

Compared with conventional text, video, graphic narratives, or generic immersive media, the principal advantage of our approach is patient-specific anatomical correspondence rather than visual intensity alone. The patient’s diagnostic chest CT is converted into a 3D representation of the individual bronchial tree, airway-lesion relationship, and anticipated bronchoscopic route, allowing procedural steps and risks to be anchored to the anatomy expected to be encountered [20,28]. The physician-operated, patient-facing interface also preserves face-to-face dialogue and allows the visualization to be navigated in response to patients’ questions, without requiring them to wear a headset or operate the software. This differs from many immersive consent interventions and extends a platform previously evaluated for bronchoscopy training to patient-facing risk communication [22,29]. These characteristics may offer advantages in personalization, spatial coherence, and integration with clinical conversation, consistent with the proposed supportive role of medical digital twins [30].

### Interpretation and Clinical Relevance

A plausible pathway is that patient-specific visualization reduces the mental translation required when unfamiliar risks are described only in words. Showing the participant’s own bronchial tree and anticipated route may provide a concrete mental model, facilitate focused questions, and increase perceived predictability. The reduction in procedure-related anxiety is compatible with this interpretation. However, outcome-related anxiety also improved even though visualization cannot resolve uncertainty about the eventual diagnosis, suggesting contributions from generalized reassurance, trust, clinician engagement, or perceived control. Patient-specific 3D and augmented-reality displays in neurosurgery have improved understanding or satisfaction, without consistently reducing anxiety [18,19]. Because this trial did not measure uncertainty, comprehension, trust, or perceived control, the mechanism remains hypothetical.

Exploratory regression plots suggested greater between-group separation among participants with higher baseline anxiety. This is plausible because those with low baseline scores had less room to improve, whereas highly anxious patients may have a greater need for individualized explanations. It should not yet guide patient selection because the analysis was not prespecified as a treatment-effect modifier analysis. Women had higher baseline VAS scores than men, but the trial was not designed to estimate sex-specific effects. Future studies should prespecify baseline anxiety, sex, prior bronchoscopy, health literacy, and preference for visual information as potential effect modifiers.

Anxiety reduction is not by itself evidence of better-informed consent. Valid consent must preserve accurate understanding of benefits, material risks, alternatives, and voluntariness [1,4,5]. Nearly all participants in both groups considered the information sufficient and helpful, while fewer digital-twin participants described it as excessive or worrying. These responses support acceptability but cannot distinguish clearer communication from false reassurance or reduced risk recall. Future evaluations should assess objective comprehension, delayed recall, decisional conflict, and risk perception. Until then, the simulator should be considered an adjunct to physician-led disclosure rather than a stand-alone consent tool.

### Implementation and Generalizability

From an implementation perspective, the intervention is most readily deployable in bronchoscopy units where diagnostic chest CT and a compatible workstation are already available. The physician-controlled display may reduce patient-side technical burden, but reconstruction time, staffing, workstation availability, and added consultation time were not prospectively measured. These constraints, together with broader challenges in digital-twin validation and workflow integration, should be assessed before scale-up [31,32].

### Strengths and Limitations

Strengths include randomized allocation across 3 hospitals, identical written information in both groups, complete intervention delivery and follow-up, and concordant findings across 2 anxiety instruments, subscales, and responder analyses. The intention-to-treat and per-protocol populations were identical, and linear mixed-effects models accounted for repeated measurements within participants.

Several limitations remain. First, this study relied exclusively on self-reported anxiety measures, including the modified APAIS and VAS, which may be subject to reporting bias. Objective physiological and behavioral indicators were not collected, and cumulative per-patient lidocaine dose and rescue analgesia were not analyzed as study outcomes. Higher satisfaction in the digital-twin group may partly reflect novelty, greater clinician attention, differences in exposure, or technology enthusiasm. Visual simulation could also increase distress in vulnerable or highly anxious patients; neither possibility was assessed prospectively. Second, consultation duration was not prospectively recorded by group, and delivery was not formally audited with audio/video recordings, independent ratings, or prospectively completed adherence checklists. Therefore, attention, exposure, and fidelity effects cannot be separated from the digital-twin component. It also remains unclear whether patient-specific CT reconstruction provides greater benefit than a standardized digital airway model. Third, only immediate preprocedural and short-term postprocedural outcomes were assessed. Fourth, although the mode and core content of disclosure were standardized, the exact number of risk items discussed with each participant was not prospectively recorded. Fifth, the exclusion of illiterate individuals and the absence of data on education, socioeconomic status, psychological history, and health literacy limit generalizability; adapted consent strategies should be evaluated for these populations. Finally, no multiplicity adjustment was applied to secondary or subscale analyses, which should therefore be interpreted as supportive and exploratory.

### Conclusions

This multicenter RCT introduces patient-specific, CT-derived bronchoscopy simulation as a risk-communication intervention, extending prior audiovisual consent research and bronchoscopy graphic-narrative work. By linking procedural explanations to each participant’s airway and lesion anatomy, the study provides evidence that individualized visualization may improve the immediate emotional experience of bronchoscopy consent, particularly among patients with greater baseline anxiety. Clinically, the approach is best considered an adjunct to, not a replacement for, physician-led disclosure. Its real-world value will depend on whether time-matched pragmatic studies confirm benefits in objective anxiety, medication use, procedural tolerance, workflow efficiency, cost-effectiveness, and in diverse or resource-constrained bronchoscopy settings.

## Supplementary material

10.2196/81382 Checklist 1 CONSORT 2025 checklist.

10.2196/81382 Checklist 2 CONSORT-EHEALTH (V 1.6.1).

10.2196/81382 Checklist 3 TIDieR checklist.

## Abbreviations

ANCOVA: analysis of covariance
APAIS: Amsterdam Preoperative Anxiety and Information Scale
CONSORT: Consolidated Standards of Reporting Trials
CONSORT-EHEALTH: Consolidated Standards of Reporting Trials of Electronic and Mobile Health Applications and Online Telehealth
CT: computed tomography
MCID: minimal clinically important difference
RCT: randomized controlled trial
TIDieR: Template for Intervention Description and Replication
VAS: visual analog scale
