# Supplementary material for: Digital Twin–Assisted Risk Disclosure in Adults Undergoing Elective Bronchoscopy: Multicenter Randomized Controlled Trial
Source: J Med Internet Res. 2026 Oct 6;28:e81382. doi: 10.2196/81382 (PMC13641757; doi:10.2196/81382)
Supplement: Supplementary file 3 [file jmir-v28-e81382-s003.pdf]

TIDieR Checklist - Completed

Digital-Twin-Assisted Risk Disclosure in Adults Undergoing Elective Bronchoscopy: Multicenter Randomized Controlled Trial

Manuscript version reviewed: August 10, 2026

Reporting note: The checklist reports both available intervention details and remaining limitations. Exact arm-specific interaction and simulator-viewing durations were not prospectively recorded, and delivery was not formally audited.

Item	TIDieR domain	Completed information	Reporting location and status
1	**Brief name**	Digital-twin-assisted risk disclosure and conventional risk disclosure.	**Reported**<br>Title; Abstract; Methods - Trial Design and Intervention and Comparator.
2	**Why: rationale and theory**	Generic text, audiovisual, and graphic consent formats have shown inconsistent effects on anxiety and usually present standardized information. Patient-specific CT reconstruction was intended to make the planned bronchoscopic route and airway-lesion relationship more concrete and personally relevant during risk disclosure.	**Reported**<br>Introduction - Background and Rationale; Objectives.
3	**What: materials**	Both groups used standardized written risk information. The intervention additionally used the Simulation AI Plus digital-twin bronchoscopy simulator (Zhejiang UE Medical Corp., China), each participant's chest CT data, a compatible workstation, and a patient-facing display. The written sheet reflected routine institutional practice and was not supplied as a separate appendix.	**Partially reported**<br>Methods - Intervention and Comparator; Figure 1. Limitation: the written information sheet is described but not reproduced in the submission.
4	**What: procedures**	All participants reviewed written information, received physician-led risk disclosure, could ask questions, and completed immediate postdisclosure anxiety assessment. In the digital-twin arm, CT data were preloaded and processed using airway segmentation, three-dimensional reconstruction, centreline extraction, and virtual bronchoscope navigation. The physician displayed the patient-specific bronchial tree, lesion relationship, and planned route, explained procedural steps and risks, and answered questions. Participants viewed but did not independently operate the simulator. The conventional arm received oral explanation and questions without simulator visualization.	**Reported**<br>Methods - Intervention and Comparator; Figure 1.
5	**Who provided**	At each center, one bronchoscopy physician trained to follow a uniform disclosure protocol delivered the assigned format.	**Reported**<br>Methods - Intervention and Comparator.
6	**How: mode of delivery**	The intervention was delivered individually and face-to-face before bronchoscopy. The physician controlled the patient-facing display; participants observed the visualization and interacted through questions rather than operating an app or simulator independently.	**Reported**<br>Methods - Intervention and Comparator.
7	**Where: setting and infrastructure**	Delivery occurred in bronchoscopy units at three hospitals in China. The digital-twin format required the participant's chest CT data to be preloaded, the Simulation AI Plus platform, a compatible workstation, and a patient-facing display.	**Reported**<br>Methods - Trial Setting and Intervention and Comparator; Figure 1.

Item	TIDieR domain	Completed information	Reporting location and status
8	**When and how much**	Each participant received one assigned prebronchoscopy disclosure session. The overall preprocedural information process, including review of written materials, typically occurred within approximately 30-40 minutes. Anxiety was reassessed immediately after disclosure and satisfaction after bronchoscopy. Exact arm-specific physician-interaction and simulator-viewing durations were not prospectively recorded.	**Partially reported**<br>Methods - Intervention and Comparator and Outcomes; Results - Intervention and Comparator Delivery. Limitation: group-specific exposure duration cannot be reported.
9	**Tailoring**	The digital-twin visualization was intentionally individualized using each participant's CT-derived airway, lesion location, and simulated bronchoscopic route. Questions were addressed individually. The conventional written material and core oral disclosure protocol were standardized.	**Reported**<br>Methods - Intervention and Comparator; Figure 1.
10	**Modifications during the study**	No changes were made to the intervention or comparator after trial commencement.	**Reported**<br>Methods - Changes to Trial Protocol.
11	**How well: planned fidelity**	Fidelity was supported by standardized written risk information, a uniform disclosure protocol, and one trained physician per center. Formal fidelity assessment using recordings, independent ratings, or prospectively completed adherence checklists was not planned or performed.	**Partially reported**<br>Methods - Intervention and Comparator; Discussion - Limitations. Limitation: no formal fidelity audit was available.
12	**How well: actual delivery**	All 122 randomized participants received their allocated disclosure intervention and completed follow-up. Because delivery was not formally audited, adherence beyond receipt of the assigned format and between-physician consistency cannot be quantified.	**Partially reported**<br>Results - Intervention and Comparator Delivery; Discussion - Limitations.

TIDieR reference: Hoffmann TC, Glasziou PP, Boutron I, et al. Better reporting of interventions: template for intervention description and replication (TIDieR) checklist and guide. BMJ. 2014;348:g1687. doi:10.1136/bmj.g1687.
